# Volatilome of Aleppo Pine litter over decomposition process

**DOI:** 10.1002/ece3.7533

**Published:** 2021-05-15

**Authors:** Justine Viros, Mathieu Santonja, Brice Temime‐Roussel, Henri Wortham, Catherine Fernandez, Elena Ormeño

**Affiliations:** ^1^ CNRS Aix Marseille Univ IRD Avignon Univ IMBE Marseille France; ^2^ Aix Marseille Univ CNRS LCE Marseille France

**Keywords:** BVOC emissions, litterbag, methanol, *Pinus halepensis*, terpenes

## Abstract

Biogenic Volatile Organic Compounds (BVOC) are largely accepted to contribute to both atmospheric chemistry and ecosystem functioning. While the forest canopy is recognized as a major source of BVOC, emissions from plant litter have scarcely been explored with just a couple of studies being focused on emission patterns over litter decomposition process. The aim of this study was to quantitatively and qualitatively characterize BVOC emissions (C_1_–C_15_) from *Pinus halepensis* litter, one of the major Mediterranean conifer species, over a 15‐month litter decomposition experiment. Senescent needles of *P. halepensis* were collected and placed in 42 litterbags where they underwent in situ decomposition. Litterbags were collected every 3 months and litter BVOC emissions were studied in vitro using both online (PTR‐ToF‐MS) and offline analyses (GC‐MS). Results showed a large diversity of BVOC (58 compounds detected), with a strong variation over time. Maximum total BVOC emissions were observed after 3 months of decomposition with 9.18 µg g_DM_^−1^ hr^−1^ mainly composed by terpene emissions (e.g., α‐pinene, terpinolene, β‐caryophyllene). At this stage, methanol, acetone, and acetic acid were the most important nonterpenic volatiles representing, respectively, up to 26%, 10%, and 26% of total emissions. This study gives an overview of the evolution of BVOC emissions from litter along with decomposition process and will thus contribute to better understand the dynamics and sources of BVOC emission in Mediterranean pine forests.

## Graphical abstract

We investigated the evolution of the BVOC emissions from the litter of *Pinus halepensis* during a 15‐month field decomposition experiment. High variability of emissions over decomposition was showed using online and offline measurements performed in vitro. Major compounds observed were monoterpenes, sesquiterpenes, and methanol, and their quantity and quality (terpenic composition) varied over sampling times with a high peak after 3 months of litter decomposition and, for a lesser extent, after 6 months for terpenic emission.

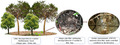

## 1 INTRODUCTION

Terrestrial ecosystems are the major source of Biogenic Volatiles Organic Compounds (BVOC) through emissions from foliage of living plants (Navarro et al., 2014) and, to a lesser extent, leaf litter (Greenberg et al., 2012). BVOC emissions have an important effect on atmospheric chemistry (Kesselmeier & Staudt, 1999) through photochemical reactions with oxidants that lead to the formation of tropospheric ozone and secondary organic aerosols (Becker et al., 1990; Griffin et al., 1999; Hoffmann et al., 1997; Ibald‐Mulli et al., 2002; Lipinski, 2011). BVOC released to the air by living plants and their litter can also impact seed germination and seedling growth of the surrounding plant species with cascading effect on plant community composition and functioning (Brilli et al., 2019; Kegge & Pierik, 2010; Ormeño et al., 2020; Santonja et al., 2019; Santoro et al., 2011).

Biogenic Volatile Organic Compounds emissions from living plants, well characterized since the 80s, consist of terpene compounds (the major class), methanol and its catabolic products (formaldehyde, formic acid), fatty acid derivatives (e.g., low chain alkanes), and phenylpropanoids including benzenoids (benzaldehyde) and simple phenolics (Gómez & Baldasano, 1999; Keenan et al., 2009; Owen et al., 1997, 2001). Factors driving their emission are also well known, with light, temperature, and water availability being the main drivers (Degenhardt et al., 2009; Genard‐Zielinski, Boissard, et al., 2015; Genard‐Zielinski, Ormeño, et al., 2014; Guenther et al., 2000; Kesselmeier & Staudt, 1999; Saunier, Ormeño, Boissard, et al., 2017; Saunier, Ormeño, Wortham, et al., 2017).

Contrastingly, little is known about BVOC emissions from plant litter, although Zimmerman et al. (1978) identified pasture litter as a potential source of BVOC. More than two decades later, Isidorov and Jdanova (2002) showed that litter of five different tree species (*Quercus robur* L., *Populus tremula* L., *Populus balsamifera* L., *Betula pendula* Rothh, *Salix* sp.) emitted both nonterpenic BVOC (e.g., alkanes, alcohols, esters), and terpenic BVOC (e.g., β‐pinene, myrcene, limonene) with contrasting emissions between species.

Biogenic Volatile Organic Compounds emissions from litter vary according to multiple factors, and the processes responsible for the emissions can either be abiotic (e.g., desorption of BVOC from the litter surface, volatilization from terpene pools such as resin ducts and glandular trichome) or biotic (e.g., microbial activity; Aaltonen et al., 2013; Leff & Fierer, 2008; Raza & Shen, 2020). Among biotic factors, litter emissions vary according to plant species identity with coniferous species (e.g., *Pinus sylvestris* L., *Picea abies* (L.) Karst) being emitters of a large variety of BVOC (terpenes, alkanes, phenols, aldehydes; Isidorov et al., 2003, 2010). The existence of secretory cavities in needles accounts for a reservoir where important amounts of terpenoids (monoterpenes and sesquiterpenes) are stored (Ormeño et al., 2009; Tang et al., 2019), partly explaining terpene emissions from needle litter. Moreover, Gray et al. (2010) showed that BVOC emissions from litter of 12 plant species (*Centaurea maculosa* Lamk., *Eucalyptus* sp., *Fraxinus pennsylvanica* Marshall, *Miscanthus* sp., *Pinus contorta* Douglas ex Loudon, *Pinus ponderosa* Douglas ex C. Lawson, *Populus deltoides* (Bartr.) Marsh., *Populus tremuloides* L., *Quercus macrocarpa* Michx., *Quercus rubra* L., *Rhododendron maximum* L., *Thinopyrum intermedium* (Host)) vary through decomposition time and attributed major emissions to biotic volatiles coming from microbial metabolism since autoclaved litter released lower emission rates. Fungi and bacteria are indeed known to release BVOC (e.g., methanol, ethanol; Misztal et al., 2018) as shown in the mVOC 2.0 database (Lemfack et al., 2018). Litter emissions are also positively driven by air temperature and litter humidity as shown for *P. ponderosa* Douglas ex C. Lawson (Greenberg et al., 2012).

*Pinus halepensis* Mill. is probably one of the main sources of BVOC emissions in coniferous forests in the Mediterranean region where it covers up to 3.5 million ha only in the Western Mediterranean region and represents 20% of the total French forest surface (208,000 ha; Simon et al., 2001). This typical Mediterranean species possesses thermophile and heliophile characteristics and is thus highly adapted to the Mediterranean climate which is characterized by relatively dry and hot summers (Rameau et al., 2008). This is also a pioneer and expansionist species (Quézel & Médail, 2003), which partly owes these features to the remarkable diversity and amounts of specific compounds it produces (terpenes and polyphenols; Chomel et al., 2014) which influence (often negatively) germination and growth of the surrounding plant species (Fernandez et al., 2013; Santonja et al., 2019). Based on the important litter amounts produced (150–530 g m^−2^ year^−1^; Arianoutsou & Radea, 2000), litter from *P. halepensis* forests could also contribute to BVOC emissions.

Only two previous studies have addressed emission of BVOC over litter decomposition. Gray et al. (2010) studied emissions from cut litter of 12 plant species incubated for 20 days at 22°C using a PTR‐MS and detected volatiles such as methanol, acetone, and terpenoids. Isidorov et al. (2010) performed measurements of *P. sylvestris* and *P. abies* litter emissions during a 490 days field litterbag decomposition experiment using Solid Phase MicroExtraction (SPME) coupled with gas chromatography‐mass spectrometry (GC‐MS) as collection and analysis techniques. This study reported quantitative and qualitative differences of terpenoid emissions through a long decomposition period.

Given the importance of BVOC emissions in both air chemistry and ecosystem functioning, the aim of the present study was to characterize (quantitatively and qualitatively) BVOC emissions from *P. halepensis* over a 15‐month field decomposition experiment using, for the first time, both, offline and online measurements. Since air and needle humidity also vary over the decomposition process, we also investigated the influence of these two abiotic factors to understand emission variability through decomposition. We hypothesized that there would be higher and more diverse emissions at the beginning of the decomposition time since terpene storage structures could provide terpene emissions.

## 2 MATERIAL AND METHODS

### 2.1 Study site and litter decomposition experiment

The study was performed in the Fontblanche forest (43°14′45.8″N, 5°39′51.2″E), located 680 m above the sea level in Southern France, (PACA‐Sud Region), where *P. halepensis* Mill. is widely spread (3,500,000 ha; Vennetier et al., 2010). This forest—managed by INRAE (Institut National de la Recherche pour l'Agriculture, l'alimentation et l'Environnement) of Avignon—is equipped with soil humidity sensors (capacitive sensor, Decagon EC‐5, Meter Environment^®^) and thermocouple T type as temperature sensors (Radiospare^®^; Table 1).

**TABLE 1:** Mean temperature and air humidity in the forest site during the litter decomposition experiment

Months	Season	Mean air temperature (°C)	Minimum air temperature (°C)	Maximal air temperature (°C)	Mean air humidity (%)	Cumulated precipitation (mm)
0–3	Autumn	10.3 ± 2.8	−2.2	23.8	66.8 ± 2.8	105.8
3–6	Winter	6.9 ± 1.6	−3.8	16.2	74.8 ± 1.3	201.8
6–9	Spring	16.5 ± 1.8	4.4	28.6	70.5 ± 2.8	242.2
9–12	Summer	22.6 ± 1	12.8	33.5	58.9 ± 1.8	102.0
12–15	Autumn	10.6 ± 1.9	−2.6	24.4	73.1 ± 4.3	500.6

Freshly fallen needles of *P. halepensis* were collected from eight individuals between mid‐July and mid‐August 2017 using litter trap nets to collect them as they fell, avoiding needles to touch the ground and stored in a thick paper bag at the ambient temperature before they were put in situ to decompose. A litterbag approach was used for litter decomposition (Santonja et al., 2015). Litterbags (15 × 15 cm, mesh size of 6 mm) filled with 17.0 g of senescent needle (15.9 g of dry weight) were placed in the Fontblanche forest floor in October 2017. Seven litterbags were collected every 3 months for a total of 5 sampling times in the field (*t* = 3, 6, 9, 12, and 15 months) in addition to an initial sampling corresponding to 7 L samples the day of litterbag deposition (*t* = 0 month). After collection, litter was stored at 4°C before being studied in the laboratory in terms of terpenic and nonterpenic emissions.

### 2.2 Litter moisture content and remaining mass

For each sampling time, litter moisture content (%) was measured as the difference between fresh and oven‐dried litter at 60°C for 3 days. Three fresh litter subsamples (of 3 g each) per litterbag were used to estimate litter moisture content. These subsamples were different from those used for volatile measurements. Remaining litter mass (g) was estimated as the total oven‐dried litter mass in each litterbag.

### 2.3 BVOC measurements

Litterbags were collected every 3 months and brought to the laboratory where they were stored at 4°C. The day before experiments, litterbags were opened, weighted and litter was let one night at room temperature. The temperature used during BVOC sampling was 30°C using a stove. Ten grams of litter were placed inside a Pyrex^®^ jar (1.2 L), while another identical jar was left empty (control). A dynamic pull–push system was used to collect BVOC released by litter and consisted of pulling up air out (0.1 L/min) from the jars while flushing an excess constant flow (0.15 L/min) of air through the jars. Air flows were regulated with mass flow controllers (MFC, 0–1 L/min, Bronkhorst). Air entering the jars was previously filtered using drierite (Hammond Company; to prevent humidity excess enter the MFC), active charcoal (Sigma‐Aldrich, untreated, mesh ≤ 5) to avoid sampling BVOC from outside the system and an ozone filter conditioned as previously described (Genard‐Zielinski, Boissard, et al., 2015; Genard‐Zielinski, Ormeño, et al., 2014; Pollmann et al., 2005; Saunier, Ormeño, Wortham, et al., 2017) to impede O_3_ reaction with BVOC within the jars. Temperature and humidity of the air inside the jars were measured using a metallic iButton^®^ Datalogger. To ensure complete removal of microorganisms, BVOC trace, and plant debris between samples, the jar was rinsed with water and then ethanol (70%), and put inside a muffle furnace at 400°C for 4 hr. Prior to the measurements, the jars were closed and conditioned at 30°C for 2 hr in a stove. The jars were then continuously flushed as previously described for 170 min. For the first 130 min, the BVOC exiting the jars were measured using a Proton Transfer Reactor–Time of Flight–Mass Spectrometer (PTR‐ToF‐MS) sequentially operated in fast‐GC and online mode, before being sampled for subsequent offline analysis for an additional time of 40 min.

### 2.4 Online BVOC collection and analysis

Online emissions measurements were carried out using a commercial PTR‐ToF‐MS (8000 instrument, Ionicon Analytik GmbH) alternately connected to the jars with and without the litter. The switch between both samples was operated by a multiposition common outlet flow path selector valve system (Vici). Mass spectra were recorded up to *m*/*z* 500 with a time resolution of one minute. The analysis was then performed when the signal was stable which corresponds to the period between 80 and 110 min after the system was connected to the analytical instrument.

The instrument was operated at a chamber pressure of 2.25 mbar, using drift tube voltage and temperature of 495 V and 380 K, respectively, corresponding to an E/N ratio (electric field strength over buffer gas number density) of ≈125 Td (1 Td = 10–17 V cm^2^). The Tofware software (version 2.5.10, TOFWERK AG; PTR module as distributed by Ionicon Analytik GmbH), running in the Igor Pro 6.3 environment (version 6.3, Wavemetrics Inc.), was used for postprocessing the mass spectra (exact mass calibration, determination of the atomic composition and fitting of the peak detected). Ion assignment relies on both fast‐GC measurements and examination of the memory effect (i.e., the time required for signal stabilization after the switching of the valve). The combination of both approaches was particularly helpful to unambiguously attribute ions to different terpenes. These species are known to produce similar product ions (Kari et al., 2018; Kim et al., 2009). Quantification was based on the online measurements. Mixing ratios were calculated using proton transfer rate constants *k* (cm^3^/s), the reaction time in the drift tube, and the experimentally determined relative ion transmission efficiency. The relative ion transmission efficiency was assessed using a standard gas calibration mixture containing 14 aromatics with molecular weight covering the range 78–180 g/mol at 100 ppb each in nitrogen (TO‐14A Aromatics Mix Restek Corporation). For the 12 species listed in 3.2, the proton transfer rate constants *k* given by Cappellin et al. (2012) at 120 Td were used. Otherwise, a default value of 2 · 10^–9^ cm^3^/s was used.

**TABLE 2:** Effects of decomposition time on measured emission compounds, remaining biomass, and mass water content of the litter. Effects are tested using Kruskal–Wallis test (K: test value)

	*K*‐value	*P*‐value
Remaining litter mass	40.62	***
Litter water content	42.79	***
Formaldehyde	41.81	***
Methanol	41.31	***
Acetaldehyde	31.89	***
Formic Acid	30.72	***
Ethanol	33.92	***
Acetone	25.59	***
Acetic Acid	37.44	***
Benzaldehyde	16.08	***
Monoterpenes	41.55	***
Oxygenated monoterpenes	39.01	***
Sesquiterpenes	40.80	***
Oxygenated sesquiterpenes	36.30	***

Note

*K*‐values and associated *p*‐values (*** for *P* < 0.001) are indicated.

Emission rates (ER) from BVOC at 30°C were calculated by considering the BVOC concentrations at the inlet and outlet of the jars using this equation:

$$\text{ER}=\frac{Q_{0}*\left(C_{\text{out}}‐C_{\text{in}}\right)}{B}$$

where ER (μg_BVOC_ g_DM_^−1^ hr^–1^, hereafter noted as μg g_DM_^−1^ hr^−1^), *Q*_0_ is the flow rate of the air introduced into the chamber (L/hr), *C*_out_ and *C*_in_ are the concentrations in the outflowing and inflowing air, respectively, (μg_BVOC_/L) and *B* is the total dry biomass matter (g_DM_).

### 2.5 Offline BVOC collection and analysis

Biogenic Volatile Organic Compounds were collected for 40 min at a flow of 0.1 L/min on glass cartridges (20 cm height; 10.5 mm external diameter) filled with Tenax TA (0.05 g, Agilent technologies, 20–35 mesh) and (0.12 g; Carbotrap Graphitized Carbon Black n°20287; Sigma‐Aldrich). These cartridges were previously conditioned for 3 hr at 300°C using a conditioner (TERA environment; RT‐12‐CN‐3601‐01) connected to a constant nitrogen flow (Nitrogen 4.5, purity 99.995%).

Analysis was performed by thermal desorption (TDS3/CIS4, Gerstel^®^) coupled to gas chromatography/mass spectrometry (GC, 6890N, MSD 5973 Agilent Technologies^®^). The BVOC trapped on the cartridges was first transferred to a cryotrap (Cooled Injection System, Gerstet) using a ramp of temperature (50°C ‐ 250°C, 3°C/s held for 15 min), at a constant flow (50 ml/min) of helium. The cryotrap was then heated from −50 to 250°C in 25 s to desorb the analytes into the capillary column (HP5‐MS, 30 m length, 0.25 mm diameter, 0.25 μm film thickness, Agilent Technologies^®^). The GC temperature program was 40°C isotherm for 5 min and up to 280°C at a rate of 3°C/min with a constant helium flow of 1.0 ml/min. The area of the different peaks in the chromatograms was used to calculate the relative contribution of the different compounds to total emissions from each class of compounds (e.g., monoterpenes, sesquiterpenes). Peak identification was carried out by using both standards of high purity (>99%, Sigma‐Aldrich^®^) and mass spectra library searches (NIST library). An additional criterion for peak identification was based on the Kovats retention index from Adams (2007).

### 2.6 Statistical analysis

We used the R software (version 3.4.X) and the agricolae package to perform the statistical analyses. Differences in remaining litter mass, litter moisture content, and BVOC emissions among the six decomposition times (i.e., from 0 to 15 months) were tested using a Kruskal–Wallis test, followed by a post hoc Fischer LSD test where significant differences are noted with different letters (a > b > c > d > e > f). Finally, Pearson correlations between the several BVOC emission variables and litter moisture were performed.

## 3 RESULTS

### 3.1 Litter mass loss and moisture content

**FIGURE 1:**
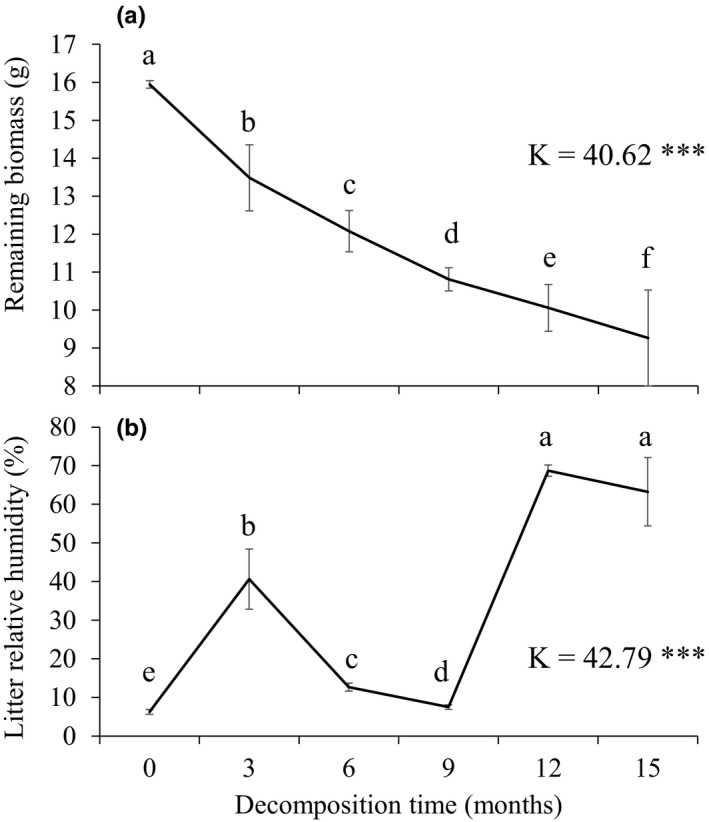
Remaining litter mass (g_DW_) (A) and litter relative humidity (%) (B) through the decomposition experiment. Values are mean ± *SD*; *n* = 7. *K*‐values (Kruskal–Wallis test) and associated *P*‐values (*** for *P* < 0.001) are indicated. Different letters denote significant differences between decomposition times with a > b > c > d > e > f (Fisher LSD post hoc tests)

Litter remaining mass inside the litterbag dropped from 15.9 g_DM_ at the beginning to 9.3 g_DM_ (42% loss) after 15 months of field decomposition (Figure 1A). The highest litter mass loss occurred during the first 3 months with a loss of 15.4%.

Litter moisture content of samples ranged from 6.2% to 68.7% over the studied period with the maximum humidity occurring in the 3rd, 12th, and 15th months of decomposition. Accordingly, the 3rd and 15th month of the experiment correspond to winter time and the 12th month to autumn time (Table 1, Figure 1B).

### 3.2 BVOC diversity

A list of 63 individual ion peaks was obtained from the investigation of the time‐resolved mass spectra provided by the PTR‐ToF‐MS (Appendix 1). Eight compounds (formaldehyde, methanol, acetaldehyde, formic acid, ethanol, acetone, acetic acid, benzaldehyde) and four compound groups (monoterpenes, oxygenated monoterpenes, sesquiterpenes, oxygenated sesquiterpenes), representing 78% of the total BVOC emission, were deeply studied to understand their evolution through the decomposition process. As a whole, this study showed a strong variation of total BVOC emission from *P. halepensis* litter both quantitatively (from 9.18 µg g_DM_^−1^ hr^−1^ after 3 months to 0.57 µg g_DM_^−1^ hr^−1^ after 12 months of decomposition) and qualitatively as the most emitted monoterpenes varied over decomposition time (Table 2). For example, terpinolene was the most emitted monoterpenes at the senescent time while α‐pinene was the most emitted monoterpenes after 6 months of decomposition.

### 3.3 Nonterpenic emissions

**FIGURE 2:**
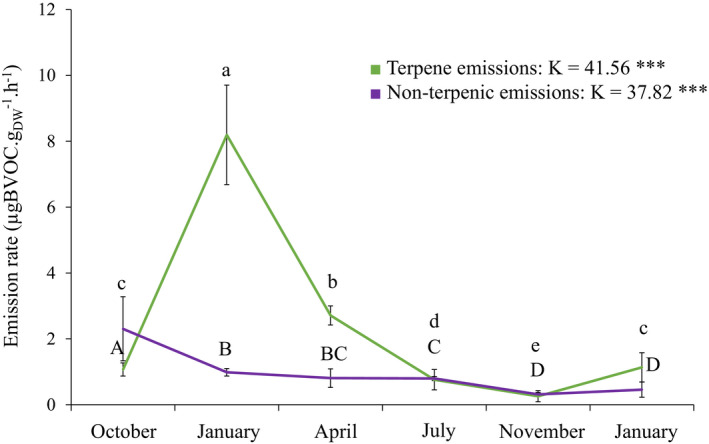
Evolution of nonterpenic (purple line) and terpenic emissions (green line) from *Pinus halepensis* litter through the decomposition experiment. Values are mean ± *SD*; *n* = 7. *K*‐values and associated *P*‐values (*** for *P* < 0.001) are indicated. Different letters denote significant differences between decomposition times with a > b > c > d > e (Fisher LSD tests)

Nonterpenic emissions were only higher than terpenic emissions at the initial stage when senescent needles had not undergone decomposition yet. At this stage, the total nonterpenic emission rate was 2.3 µg g_DM_^−1^ hr^−1^ and declined progressively up to 0.5 µg g_DM_^−1^ hr^−1^ after 15 months of decomposition (Figure 2).

**FIGURE 3:**
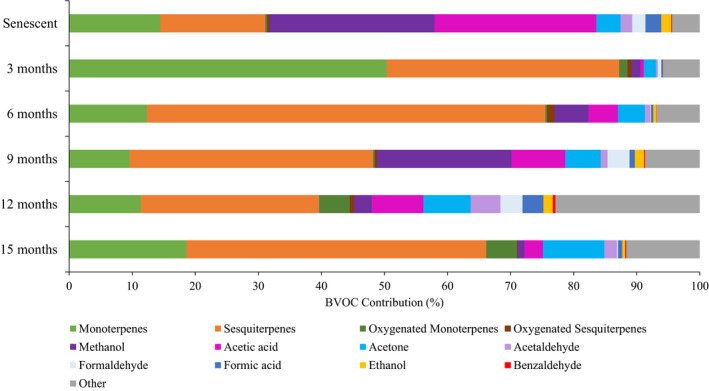
Contribution (%) of the twelve major BVOC to the total emissions across the six sampling times (from 0 to 15 months of decomposition). Values are mean; *n* = 7

**FIGURE 4:**
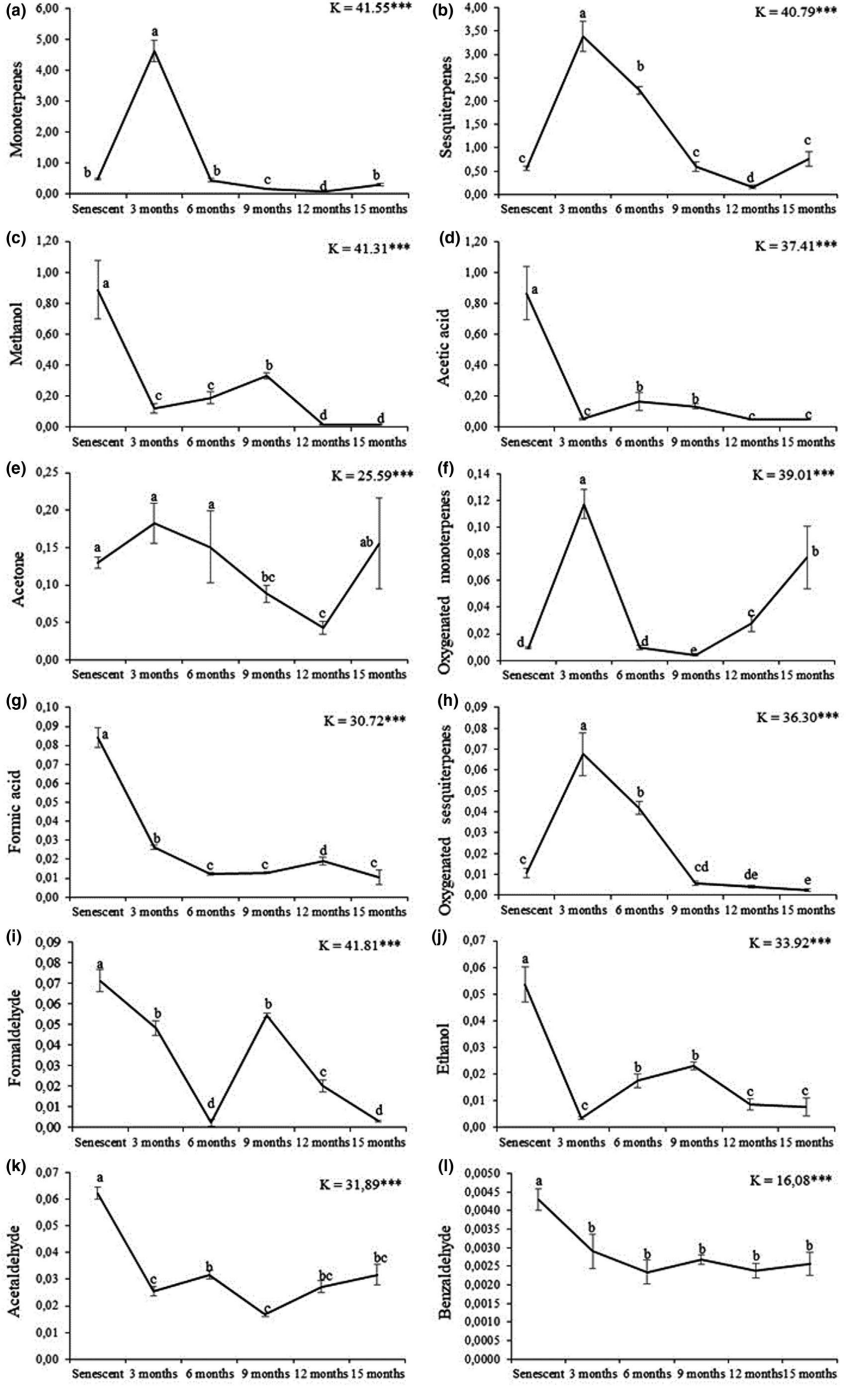
Litter emission rates (µg g_DM_^−1^ hr^−1^) of the 12 BVOC emitted across the six sampling times (from 0 to 15 months of decomposition). Values are mean ± *SE*; *n* = 7. *K*‐values (Kruskal Wallis test) and associated *P*‐values (*** for *P* < 0.001) are indicated. Different letters denote significant differences between decomposition times with a > b > c > d > e (Fisher post‐hoc LSD tests)

There were also important qualitative differences through the decomposition process although the three most emitted compounds were always methanol, acetic acid, and acetone (Figure 3; Appendix 2 and 3). Methanol was the most emitted compound from *P. halepensis* senescent needles (0.89 µg g_DM_^−1^ hr^−1^). Its emission declined rapidly after 3 months of decomposition (0.12 µg g_DM_^−1^ hr^−1^), showed a slowly rise after 9 months, and declined thereafter until negligible emission rates (<0.01 µg g_DM_^−1^ hr^−1^; Figure 4C, Table 2). Ethanol showed a similar pattern as methanol although it exhibited smaller emission rates (Figure 4J). Likewise, acetic acid showed its highest emission rate for senescent needles (0.87 µg g_DM_^−1^ hr^−1^) and lower emissions after 3, 12, and 15 months of decomposition (0.05 µg g_DM_^−1^ hr^−1^) (Figure 4D). Variations of acetone emission contrasted to the compound cited above, with maximum emission rates after 3, 6, and 15 months (0.18, 0.15, and 0.16 µg g_DM_^−1^ hr^−1^, respectively) and the lowest emission rate after 12 months of decomposition (0.04 µg g_DM_^−1^ hr^−1^; Figure 4E). Also, formaldehyde peaked after 6 months of decomposition while similar moderate emissions were recorded at 3 and 9 months of decomposition (~0.05 µg g_DM_^−1^ hr^−1^ each; Figure 4I). Benzaldehyde exhibited the lowest emission rates with emissions lower than 0.005 µg g_DM_^−1^ hr^−1^ (Figure 4L).

### 3.4 Terpenic emissions

Terpenic compounds were the most emitted compounds and contributed from 71% to 89% of the total emissions after 3, 6, and 15 months of decomposition. After 9 and 12 months, terpene and nonterpene emissions were similar (Figures 2 and 3; Appendix 2 and 3). Senescent needles represented 32% of total emission. Nonoxygenated monoterpenes and sesquiterpenes represented the major terpenic fraction.

**FIGURE 5:**
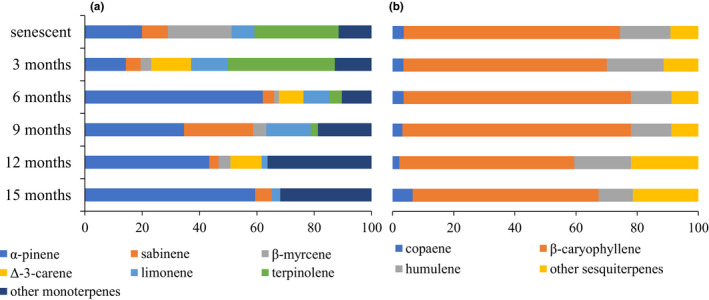
Contribution (%) of the major monoterpenes to the total monoterpene emissions (*m*/*z* 136) (a) and contribution of the major sesquiterpenes to total sesquiterpene emissions (*m*/*z* 204) (b) across the six sampling times (from 0 to 15 months of decomposition)

Monoterpenes featured the highest emission rates after 3 months of decomposition (4.63 µg g_DM_^−1^ hr^−1^), lower emissions at senescent stage, 6, 9, and 15 months of decomposition (from 0.15 to 0.49 µg g_DM_^−1^ hr^−1^), and negligible emission at 12 months of decomposition (< 0.1 µg g_DM_^−1^ hr^−1^) (Figure 4A). The emitted monoterpenes profile also changed along with the decomposition. The six most emitted monoterpenes were α‐pinene, terpinolene, Δ‐3‐carene, limonene, sabinene, and β‐myrcene. After 3 months of decomposition, emissions were characterized by a high proportion of terpinolene. α‐pinene occurred all along the decomposition process and was the major monoterpene after 6, 12, and 15 months of decomposition (Figure 5a).

Major (nonoxygenated) sesquiterpene emissions occurred after 3 and 6 months decomposition (3.38 and 2.22 µg g_DM_^−1^ hr^−1^, respectively) while lower emissions were recorded at senescent stage, 9, 12, and 15 months of decomposition (from 0.76 to 0.16 µg g_DM_^−1^ hr^−1^) (Figures 3 and 4B). Major sesquiterpenes were β‐caryophyllene (from 57% to 75% of total sesquiterpenes), α‐humulene (13%–19%), and copaene (2%–7%) (Figure 5b).

Oxygenated monoterpenes showed higher emission rates after 3 months of decomposition (0.12 µg g_DM_^−1^ hr^−1^) compared with senescent needles and later decomposition times. Emissions after 12 and 15 months of decomposition showed a slightly increasing emission (0.03 and 0.08 µg g_DM_^−1^ hr^−1^, respectively).

Oxygenated sesquiterpenes showed the highest emission rates after 3 and 6 months of decomposition (0.68 and 0.42 µg g_DM_^−1^ hr^−1^, respectively), while negligible emission were monitored during the rest of the experiment (≤0.01 µg g_DM_^−1^ hr^−1^) (Figure 4H).

### 3.5 Relationships between compound emissions rates

**FIGURE 6:**
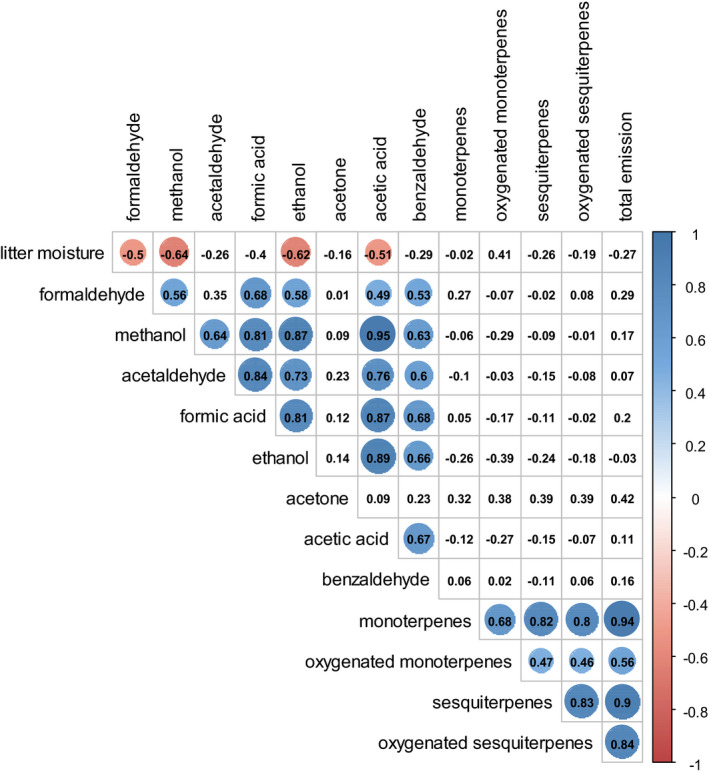
Correlation matrix (Pearson) between BVOC emission variables and litter moisture. Red color indicates significant negative correlation, while blue color indicates significant positive correlations (*P* < 0.05). *n* = 42. Volatiles are ranged according to increasing *m*/*z*

Some volatiles (formaldehyde, methanol, ethanol, acetic acid) were negatively correlated with litter moisture content (from *r* = −0.64 to *r* = −0.50). Strong correlations were observed between the emissions of highly volatile compounds (Figure 6), such as for example between methanol and ethanol (*r* = 0.87), methanol and acetic acid (*r* = 0.95), acetaldehyde and formic acid (*r* = 0.84), or ethanol and acetic acid (*r* = 0.89). Also, strong correlations were found between terpenic compounds (Figure 6), such as between monoterpenes and sesquiterpenes (*r* = 0.82) or between sesquiterpenes and oxygenated sesquiterpenes (*r* = 0.83). More importantly, the total emission was highly correlated to terpenic emission and specifically to monoterpenes (*r* = 0.94) and sesquiterpenes (*r* = 0.90).

## 4 DISCUSSION

Our study showed that BVOC emissions from *P. halepensis* litter evolved both qualitatively and quantitatively during the decomposition process (from 0 to 15 months). Dynamics of BVOC emission over the decomposition period were probably the result of different combined processes which account for sinks and sources. The sources of BVOC are diverse, as volatiles could be directly released from litter tissues (Peñuelas et al., 2014; Viros et al., 2020), or result from microbial activity during litter consumption (Gray et al., 2010; Isidorov et al., 2010) and microbial emissions (Effmert et al., 2012; Lemfack et al., 2018). Adsorption of some volatiles on litter surface and dissolution in water according to litter humidity can act as possible sinks of BVOC (Insam & Seewald, 2010; Seco et al., 2008). Moreover, microorganisms could also act as a sink through their consumption of highly volatile compounds (Chaignaud et al., 2018; Morawe et al., 2017). These potential processes are hereafter discussed for terpenic and nonterpenic compounds separately. After 15 months of decomposition, all emissions (including those of highly volatile compounds) exhibited negligible emission rates. Such time of decomposition corresponds to a high decomposition degree since we recorded a 42% mass loss after 15 months of decomposition and previous experiments on *P. halepensis* litter decomposition reported 53% litter mass loss after 3 years of decomposition in Southern France (Chomel et al., 2014). Chadwick et al. (1998) reported that *P. sylvestris* had lost from 20% to 35% of its original litter mass after 7 months of decomposition, a finding close to the present study where we observed 25% of decomposition over a similar decomposition period. Moro and Domingo (2000) measured that litter from *Pinus nigra* Arnold and *Pinus pinaster* Aiton had lost 20 to 25% of its original mass after 1.5 year of decomposition.

### 4.1 Emissions of terpenic compounds

As with other coniferous species, *P. halepensis* possesses secretory cavities in green needles and consequently in needle litter where terpenes are stored in very high amounts (up to several tens of mg/g_DM_) (Ormeño et al., 2009; Valor et al., 2017). During decomposition, the alteration of needle structure is favoring terpene exchange from terpene pools to the atmosphere. Since senescent needles have not undergone decomposition yet their needle structure is intact. The result is a rather low terpene emission rate at the beginning of the decomposition process. By contrast, after 3 months of decomposition we recorded the greatest terpenic emissions (8.19 µg g_DM_^−1^ hr^−1^) which accounted for 89% of total emission. Cell walls of the needles tissues were presumably partly degraded after 3 months, strongly favoring BVOC emissions from needle terpene pools to the air. Since fungi can also emit some terpenic compounds such as limonene or camphor, we cannot exclude that some emissions also originate from microorganisms (Effmert et al., 2012). However, since litter terpene emissions exhibited high correlations between them, it is likely that most terpenes originate from needle terpene pools. The determination of the presence of microorganisms using phospholipid‐derived fatty acids technics (PLFA) and their potential emission using the mVOC database (Lemfack et al., 2018) could help understand the contribution of the microbial emission to the total emission of the litter.

Six monoterpenes, including α‐pinene, terpinolene, Δ‐3‐carene, limonene, sabinene, and myrcene, were detected in most of the emissions and represented the major part of the global emission. Litter of *Pinus* spp. stores (Ormeño et al., 2009) and releases these compounds (Isidorov et al., 2003; Mali et al., 2019). Kainulainen and Holopainen (2002) showed that monoterpenes stored in *P. sylvestris* litter decreased by 68% during the first 7 months of decomposition, showing that terpenes contained in the terpene storage pool were progressively emptied along with the decomposition process. They also showed that some monoterpenes (e.g., myrcene, limonene) were emitted before others (e.g., α‐pinene) which can potentially explain the different monoterpene composition between the decomposition times reported in the present study.

Oxygenated monoterpenes only represented a minor fraction of the total terpenic emission. Their emission can be mostly attributed to the same processes described before, being desorbed from the terpene pool. However, some oxygenated monoterpenes such as camphor have also been reported to be emitted by fungi and bacteria (Effmert et al., 2012).

In this work, sesquiterpenes reached the highest emission rates after 3 and 6 months of sampling times (3.38 and 2.22 µg g_DM_^−1^ hr^−1^, respectively). Sesquiterpenes emissions were mostly represented by β‐caryophyllene, α‐humulene, and copaene. Caryophyllene, being the most dominant sesquiterpene emitted (from 57% to 75% of the total emissions of sesquiterpenes), is also the most emitted sesquiterpene from Amazonian soil in aerobic conditions (Bourtsoukidis et al., 2018). This great presence of sesquiterpenes on a longer time scale compared with monoterpenes can be explained by their moderate volatility (Tang et al., 2019). This condition could be identified as a slower volatilization of the sesquiterpenes contained in the resin ducts requiring a longer exposition at the decomposition process to sufficiently alter the needle structure and fully expose these compounds to the ambient air. Oxygenated sesquiterpenes contributed insignificantly to total terpenic emission.

### 4.2 Emission of nonterpenic compounds

Highly volatile nonterpenic compounds represented the major emissions at the senescent needle stage (68%) and accounted for half of the emission after 9 and 12 months of decomposition.

Methanol was the main highly volatile BVOC, in agreement with previous studies focusing on emissions from bare soil and litter of *Pinus taeda* L. (Bachy et al., 2018; Ramirez et al., 2010; Tang et al., 2019). Accordingly, ecosystem‐scale methanol emissions have been suggested to reach the highest fluxes in summer due to litter contribution in a *P. ponderosa* forest in California (Bouvier‐Brown et al., 2012). Although Gray et al. (2010) also measured important methanol emissions from incubated litter in a laboratory experiment, they represented 95% of total emissions contrasting with our study. Litter cut and drying in Gray et al. (2010) before BVOC collection probably explain this difference. In our experiment, methanol emissions were the highest at the initial decomposition stage (senescent needle). Accordingly, methanol emissions are also released from plant cell wall (McFeeters & Armstrong, 1984). Since senescent litter has not been in contact with soil yet, methanol emissions can easily reach the atmosphere. As saprophytes progressively colonize litter, methanol is rapidly consumed by methanol‐oxidizing prokaryotes since this is the main labile compound, creating a methanol sink (Abanda‐Nkpwatt et al., 2006; Chaignaud et al., 2018; Kolb, 2009; Morawe et al., 2017). It should however be acknowledged that methanol can also be emitted by microorganisms (Gray et al., 2010; Schink & Zeikus, 1980; Schulz & Dickschat, 2007). We hypothesize that the methanol peak after 9 months of decomposition is again the result of emissions related to ongoing cell wall degradation associated with a negligible methanol consumption by aerobic microorganisms.

Formaldehyde emissions varied over decomposition time. Variations could be partially explained by changes in litter moisture since this is a highly water‐soluble compound (Dong & Dasgupta, 1986). Accordingly, there was a negative correlation between formaldehyde emission and litter water content. Small amounts of formaldehyde can also be produced by saprophytic fungi such as *Ceratocystis* spp. (Effmert et al., 2012; Stotzky et al., 1976). Some studies previously reported a major uptake of formaldehyde by soils due to dissolution or absorption (Dong & Dasgupta, 1986; Gray et al., 2014; Tang et al., 2019). However, further studies are required to understand whether soil (surface + litter) is a net source or sink of formaldehyde under different soil humidity conditions.

In this study, acetaldehyde was a small part of the total BVOC emission from *P. halepensis* litter. Gray et al. (2010) measured strong emissions of acetaldehyde from autoclaved cut litter and attributed such response to the nonbiotic part of emission (i.e., cell wall decomposition, catabolic product of degradation from other compounds). Past studies have showed that acetaldehyde originates from both the organic matter decomposition (carbohydrates and proteins) and oxidation of ethanol (Tang et al., 2019). Supporting this last hypothesis, acetaldehyde, and ethanol emissions showed a strong relationship (*r* = 0.73).

Acetic and formic acids, the most emitted acids from plants (Kesselmeier & Staudt, 1999), were also emitted by litter, as showed in our study, with low emission rates (0.05–0.98 µg m^−2^ hr^−1^) contrasting to foliage emissions of living trees, shrubs, and crops (35 µg m^−2^ hr^−1^; Paulot et al., 2011). *Acetobacteraceae*, from which 10 genera produce acetic acid, is found to be one of the most encountered bacteria family in the senescent stage and during the early stages of decomposition of *P. sylvestris* litter as this family of bacteria is often reported as early decomposer (Gołębiewski et al., 2019). Furthermore, formic acid is emitted by fungi (Effmert et al., 2012; Wheatley et al., 1996) and bacteria (Effmert et al., 2012; Stotzky et al., 1976) and is also the result of formaldehyde oxidation (Andrushkevich et al., 2014). Acetic acid emissions were only important at the senescent needle stage when they accounted for 26% of total emissions. Mozaffar et al. (2018) showed that senescent *Zea mays* L. leaves still attached to the plant released acetic acid represented up to 30% of total emission recorded just before the abscission. A recent study showed that senescent leaves from *Populus* spp. might produce an outburst of oxygenated BVOC such as methanol and acetic acid (Portillo‐Estrada et al., 2020). The biogenic fraction of formic and acetic acid emission is found to be accounted for 55 to 100% of the global acid emission (Glasius et al., 2001), representing a major source, litter is here found to be a small but existent portion of it. These acids seemed to result from methanol oxidation as suggested by Schade and Goldstein (2001), which is supported by the very high correlation between methanol and these compounds in the present study (Figure 6).

Several studies have detected ethanol emissions from *Fagus* sp., *Quercus* sp., *Picea* sp. and *Pinus* sp. leaf and needle litter (Isidorov et al., 2003; Warneke et al., 1999). It is likely that the few amounts detected originate from associated bacterial and fungal metabolism (Effmert et al., 2012). Succession of microbial communities during the decomposition associated with decomposing litter itself could explain emission variations through the study period. Moreover, ethanol oxidation results in formation of acetaldehyde and acetic acid (Kreuzwieser et al., 1999), so results found in our study are probably the combined effect of both processes. Acetone has also been detected in emissions from *Fagus* sp., *Quercus* sp. and *Picea* sp. leaves and needles litter (Greenberg et al., 2012; Warneke et al., 1999). Warneke et al. (1999) reported that combination of heat and humidity can produce large amounts of acetone, but litter emits less acetone than canopy. Gray et al. (2010) found acetone to contribute to the emission of less than 3% when both biotic and abiotic emission were measured to about 10% of abiotic global emission from litter of *P. ponderosa* autoclaved litter. In our study, acetone represents from 2% to 10% after 3 and 15 months of decomposition, respectively.

Finally, only very few emissions of benzaldehyde were measured making it negligible in the global litter BVOC emissions. Isidorov and Jdanova (2002) showed emissions of benzaldehyde from all the leaf litters studied, but this compound was not quantified. Benzaldehyde emissions from green leaves in canopy were often attributed to a different source than plants or canopy. Owen et al. (1997) measured benzaldehyde along with other aldehydes but were not certain of its origin. Kesselmeier et al. (2000) measured benzaldehyde on the atmosphere within the tropical forest but partly attributed this emission to the activity of bacteria or fungi.

### 4.3 BVOC emission from litter and its implication on the environment

Numerous studies focusing on BVOC emissions from the canopy and green leaves have shown the ecophysiological role of BVOC released by one species on the surrounding plant species. For example, Ormeño et al. (2020) showed that isoprene emission confers oxidative protection on *Acer monspessulanum* leaves. de Boer et al. (2019) showed pathogen suppression in soil by BVOC emissions. Garbeva and Weisskopf (2020) demonstrated that BVOC released by bacteria can have a significant influence on the plant health as it can, for example, induce a growth stimulation of *Arabidopsis thaliana* L. BVOC emissions from litter and associated microbial communities can also greatly influence plant species performances and soil biota with cascading effects on plant community composition and ecosystem processes. For example, Santonja et al. (2019) showed that BVOC emissions from *P. halepensis* needle litter inhibit seed germination and seedling growth of two herbaceous species. β‐caryophyllene was pointed out to explain this allelopathic effect of BVOC. *P. halepensis* being a large producer of this compound it has thereby the potential to greatly impact the surrounding plant species composition. In addition, litter terpene content has also been shown to slow down the litter decomposition process (Chomel et al., 2016) by impacting the soil microbial community structure (Santonja et al., 2018) and the whole soil food web (Chomel et al., 2016).

With the ongoing climate change, a stronger attention needs to be paid to litter BVOC emissions that are strongly influenced by climatic conditions. In the present study, we showed that air temperature controls both terpenic and nonterpenic compound emissions (Appendix 4), probably due to the volatilization of the terpenes stored with increasing temperature. Previous studies also demonstrated that the air temperature controls BVOC emissions from green leaves (Guenther et al., 1993). Likewise, Greenberg et al. (2012) also showed a dependence of litter BVOC emissions of a coniferous species (*P. ponderosa*) with air temperature and soil moisture.

### 4.4 Litter BVOC emission role in biosphere and atmosphere exchanges

The influence of BVOC emission from litter and soil in the global atmospheric oxidative budget is still not well known. Their importance varies from one compound to another, and several studies show that terpenic emissions are more likely to react rapidly with the atmospheric oxidants such as O_3_, NO_3_, and the OH· radical but each terpenic compound reacts differently (Atkinson, 2000; Guenther et al., 1995). For example, the terpenes that were found in our study are involved in the production of secondary organic aerosols (Hartz et al., 2005). According to Lee et al. (2006), α‐pinene, β‐caryophyllene, and α‐humulene have a high SOA mass yield (41%, 45%, and 45%, respectively), while terpinolene has a smaller SOA mass yield (20%). These compounds, as most terpenes in comparison with nonterpenic compounds, are also highly reactive with OH· radical (Atkinson & Arey, 2003) revealing the importance of terpenes in atmospheric chemistry. Nonterpenic emissions also influence the secondary air pollution and the oxidizing capacity of the atmosphere. Acetone and methanol, when oxidized, lead to the formation of OH (Folkins & Chatfield, 2000; Mari et al., 2002). Formic and acetic acids contribute to the rain acidity (Andreae et al., 1988; Talbot et al., 1990). Also, short chained aldehydes have been proven to play an important role in the troposphere oxidative budget (Carlier et al., 1986; Singh et al., 2001). The oxidation of some of these nonterpenic compounds can also lead to the formation of additional products, for example, methanol can be oxidized to formaldehyde (Alwe et al., 2019; Singh et al., 2000). However, it is important to note that the presence of these highly volatile compounds is always depending on the balance between source and sink of BVOC (Kuhn et al., 2002; Paulot et al., 2011; Rottenberger et al., 2004; Trowbridge et al., 2020). When compared to emissions of BVOC from green leaves of *P. halepensis* that vary from one study to another with a range of emissions between 3.0 and 15.0 µg g_DW_^−1^ hr^−1^ (Llusia & Penuelas, 2000; Ormeño et al., 2007), our study shows that emission from *P. halepensis* litter can highly contribute to the total emissions in *P. halepensis* forests, with a major contribution after 3 and 6 months of decomposition. Thus BVOC emission from litter could extend the seasonal period of BVOC emission to winter and thereby probably extend the biogenic participation of BVOC to SOA formation (Faiola et al., 2014). Also, in the Mediterranean region, litter production of *P. halepensis* is moderate (150–530 g m^−2^ year) but this species is covering an important surface of the Western Mediterranean region (~3.5 million ha; Quézel & Médail, 2003). The estimation of the potential ozone and SOA formation (POCP and SOAP, respectively) of the senescent litter of *P. halepensis* has been calculated in a recent study (Viros et al., 2020) showing the predominance of terpenes to highly contribute to the formation of these secondary pollutants. In our study, we showed that the presence of monoterpenes and sesquiterpenes is even higher at 3 and 6 months of decomposition than at the senescent time which could imply an even higher potential of ozone and SOA formation through emissions from decomposed litter. Litter of *P. halepensis* could thereby importantly contribute to BVOC emissions and atmospheric chemistry in the Mediterranean region.

## 5 CONCLUSION

Although green leaves are known as the main source of BVOC emissions, *P. halepensis* litter has been proved herein to be a non‐negligible source of BVOC especially at the beginning of the decomposition process. The highest emission rates were observed at 3 months of decomposition (9.18 µg g_DM_^−1^ hr^−1^) and both terpenic emissions (e.g., monoterpenes, sesquiterpenes) and nonterpenic emissions (e.g., methanol, acetic acid) evolved over the decomposition process.

However, this is a preliminary study performed without soil and at 30°C, while BVOC emission measurements using litter on soil would probably result in lower emission rates since soil is a sink of BVOC (Chaignaud et al., 2018; Paulot et al., 2011; Rinnan & Albers, 2020). Lower temperatures would also probably diminish the emission rates but extend the emission period (Greenberg et al., 2012; Grote & Niinemets, 2008). Future research performed both in the field and the laboratory, using soil and litter together or isolated would allow to estimate the global balance of BVOC emission and uptake from both compartments on a soil humidity and air temperature range. Future studies could also be conducted to elucidate whether different microbial communities colonizing litter modify the profile of litter BVOC emissions. Finally, it could also be interesting to deeply link litter BVOC emission to degradation needle structures using Scanning Electron Microscopy (SEM) as a tool to visualize the timing when secretory cavity degradation is enough to favor BVOC release to the atmosphere. Such studies would contribute to understand which are the major factors influencing BVOC litter emission.

## CONFLICT OF INTEREST

None declared.

## AUTHOR CONTRIBUTIONS

**Justine Viros:** Data curation (lead); Formal analysis (lead); Investigation (lead); Methodology (lead); Writing‐original draft (lead); Writing‐review & editing (lead). **Mathieu Santonja:** Formal analysis (equal); Writing‐review & editing (equal). **Brice Temime‐Roussel:** Data curation (supporting); Formal analysis (supporting); Investigation (equal); Resources (equal); Software (supporting); Writing‐review & editing (supporting). **Henri Wortham:** Conceptualization (equal); Resources (equal); Writing‐review & editing (equal). **Catherine Fernandez:** Conceptualization (lead); Funding acquisition (lead); Project administration (equal); Supervision (equal); Writing‐review & editing (equal). **Elena Ormeno:** Conceptualization (lead); Funding acquisition (lead); Project administration (equal); Resources (equal); Supervision (lead); Writing‐original draft (lead); Writing‐review & editing (equal).

## Data Availability

The data supporting this article are available from the Dryad Digital Repository (https://doi.org/10.5061/dryad.sf7m0cg5w).

## APPENDIX 1

List of ions detected by the PTR‐ToF‐MS (for conciseness, corresponding ^13^C isotopic ions have been omitted).

**Table:** Note

Formula	Assignment	*m*/*z*	k rate (10^–9^ cm^3^/s)	Type of identification
(CH_2_O)H+	Formaldehyde	31.02		Compound
(CH_3_OH)H+	Methanol*	33.03	2.22	Compound
C_3_H_5_+	Isoprene fragment	41.04		
(C_2_H_3_O)H+	Acetic acid fragment	43.02		
(C_2_H_4_O)H+	Acetaldehyde*	45.03	3.12	Compound
(CH_2_O_2_)H+	Formic Acid*	47.01	1.99	Compound
(C_2_H_6_O)H+	Ethanol*	47.05	2.18	Compound
(C_3_H_4_O)H+	Acrolein	57.03		Compound
(C_3_H_6_O)H+	Acetone*	59.05	3.32	Compound
(C_2_H_4_O_2_)H+	Acetic acid*	61.03	2.22	Compound
(C_5_H_8_)H+	Isoprene/monoterpene fragment	69.07	1.7	Compound
(C_4_H_6_O)H+	Methacroleine/MVK*	71.05	3.3	Compound
(C_3_H_4_O_2_)H+	Methylglyoxal	73.03		Compound
(C_4_H_8_O)H+	Butanal/methylpropanal*	73.06	3.2	Compound
(C_6_H_6_)H+	Aromatic	79.05		Compound group
C_6_H_9_+	Monoterpene fragment	81.07		Compound group
(C_5_H_6_O)H+	Methyl furan	83.05		Compound group
C_6_H_11_+		83.09		Compound group
(C_5_H_8_O)H+		85.06		Compound group
(C_4_H_6_O_2_)H+		87.04		Compound group
(C_5_H_10_O)H+	Pentanone/pentanal	87.08	3.24	Compound group
(C_4_H_9_O_2_)H+	Ethyl acetate	89.06		Compound group
(C_7_H_8_)H+	Toluene/Monoterpene fragment	93.07		Compound
C_7_H_11_+	Terpene fragment	95.09		Compound group
(C_7_H_12_)H+		97.10		Compound group
(C_6_H_8_O)H+		97.06		Compound group
(C_5_H_6_O_2_)H+		99.04		Compound group
(C_6_H_10_O)H+		99.08		Compound group
(C_5_H_8_O_2_)H+		101.06		Compound group
(C_6_H_12_O)H+	Hexanone/hexanal	101.10		Compound group
(C_5_H_10_O_2_)H+		103.08		Compound group
(C_8_H_8_)H+		105.07		Compound group
(C_4_H_6_O)H+	Benzaldehyde	107.05		Compound
C_8_H_11_+	Terpene fragment	107.09		
(C_8_H_12_)H+	Sesquiterpene fragment	109.10		
(C_7_H_10_O)H+		111.08		Compound group
(C_8_H_14_)H+		111.12		Compound group
(C_6_H_8_O_2_)H+		113.06		Compound group
(C_6_H_13_O)H+		113.10		Compound group
(C_6_H10O_2_)H+		115.08		Compound group
(C_6_H_12_O_2_)H+	Ethyl butanoate/Butyl acetate	117.09		Compound group
(C_9_H_10_)H+		119.09		Compound group
C_9_H_13_+	Terpene fragment	121.10		
C_9_H_15_+	Terpene fragment	123.12		
(C_8_H_12_O)H+		125.10		Compound group
(C_9_H_16_)H+		125.13		Compound group
(C_6_H_6_O_3_)H+		127.04		Compound group
(C_7_H_10_O_2_)H+		127.08		Compound group
(C_8_H_14_O)H+		127.11		Compound group
(C_10_H_12_)H+		133.10		Compound group
(C_10_H_14_)H+	p‐cymene/terpene fragment	135.12		Compound
(C_10_H_16_)H+	Monoterpene	137.13	2.5	Compound group
				
(C_9_H_14_O)H+	Nopinone	139.11		Compound
(C_11_H_14_)H+		147.12		Compound group
C_11_H_17_+	Sesquiterpene fragment	149.13		
(C_10_H_14_O)H+	Oxygenated monoterpene	151.11		Compound group
(C_10_H_16_O)H+	Oxygenated monoterpene	153.13		Compound group
(C_10_H_20_O)H+	Oxygenated sesquiterpene	157.16		Unknown
(C_9_H_18_O_2_)H+		159.02		Compound group
C_12_H_19_+	Sesquiterpene fragment	163.15		
(C_13_H_20_)H+		177.16		Compound group
(C_15_H_24_)H+	Sesquiterpene	205.20	3	Compound group
(C_15_H_24_O)H+	Oxygenated sesquiterpene, sesquiterpene	221.19	2	Compound group

*k*: proton transfer rate constant (cm^3^/s).

## APPENDIX 2

Contribution (%) of the major nonterpenic BVOC to the total emission across the six sampling times (from 0 to 15 months of decomposition). Values are mean ± *SD*, *n* = 7.

**Table:**

Time of decomposition	Formaldehyde	Methanol	Acetaldehyde	Formic acid	Ethanol	Acetone	Acetic acid	Benzaldehyde	Other
Senescent	2.11 ± 0.42	26.26 ± 14.61	1.84 ± 0.18	2.49 ± 0.42	1.59 ± 0.51	3.84 ± 0.56	25.62 ± 13.36	0.13 ± 0.02	4.40 ± 0.79
3 months	0.52 ± 0.10	1.30 ± 0.83	0.28 ± 0.05	0.29 ± 0.03	0.04 ± 0.01	1.98 ± 0.78	0.56 ± 0.11	0.03 ± 0.01	5.75 ± 1.07
6 months	0.07 ± 0.13	5.35 ± 2.86	0.90 ± 0.12	0.35 ± 0.05	0.50 ± 0.19	4.29 ± 3.59	4.67 ± 4.56	0.07 ± 0.02	6.81 ± 0.56
9 months	3.49 ± 0.20	21.31 ± 3.18	1.07 ± 0.1	0.83 ± 0.05	1.48 ± 0.24	5.64 ± 1.97	8.51 ± 3.00	0.17 ± 0.02	8.66 ± 2.95
12 months	3.50 ± 1.93	2.81 ± 1.73	4.73 ± 1.44	3.33 ± 1.17	1.49 ± 1.36	7.47 ± 5.28	8.18 ± 1.06	0.41 ± 0.13	22.89 ± 6.61
15 months	0.19 ± 0.05	1.05 ± 0.54	1.99 ± 0.64	0.66 ± 0.62	0.47 ± 0.55	9.73 ± 10.03	2.95 ± 0.69	0.16 ± 0.05	11.66 ± 3.96

## APPENDIX 3

Contribution (%) of the terpenic BVOC to the total emission across the six sampling times (from 0 to 15 months of decomposition). Values are mean ± *SD*, *n* = 7.

**Table:**

Time of decomposition	Monoterpenes	Oxygenated Monoterpenes	Sesquiterpenes	Oxygenated Sesquiterpenes
Senescent	14.48 ± 2.15	0.28 ± 0.04	16.65 ± 3.89	0.32 ± 0.17
3 months	50.38 ± 10.12	1.28 ± 0.32	36.85 ± 9.16	0.74 ± 0.30
6 months	12.35 ± 4.15	0.28 ± 0.13	63.17 ± 6.12	1.19 ± 0.24
9 months	9.60 ± 2.75	0.26 ± 0.12	38.61 ± 17.17	0.37 ± 0.16
12 months	11.39 ± 6.97	4.83 ± 3.83	28.26 ± 24.29	0.72 ± 0.38
15 months	18.59 ± 8.13	4.83 ± 3.87	47.55 ± 24.89	0.16 ± 0.09

## APPENDIX 4

Effects of climatic variations during the decomposition process on the BVOC emissions. The significative values (*p*‐values < 0.05) are indicated with a bold font.

**Table:**

	Total terpenic emissions			Total nonterpenic emission			Total emissions		
	Intercept	Slope	*r*	Intercept	Slope	*r*	Intercept	Slope	*r*
Temperature (mean)	**6.16**	**−0.26**	**−0.56**	**1.07**	**−0.03**	**−0.61**	**7.23**	**−0.29**	**−0.58**
Temperature (minimum)	**3.05**	**−0.23**	**−0.56**	**0.71**	**−0.03**	**−0.62**	**3.76**	**−0.26**	**−0.58**
Temperature (maximum)	**8.17**	**−0.22**	**−0.47**	**1.39**	**−0.03**	**−0.58**	**9.56**	**−0.25**	**−0.49**
Soil humidity	−3.64	0.09	0.19	**−1.02**	**0.02**	**0.51**	−4.67	0.11	0.23
Precipitations	3.34	−0.005	−0.25	0.66	−0.00016	−0.08	3.99	−0.005	−0.24
